# *IL*-*17F* gene polymorphism is associated with susceptibility to acute myeloid leukemia

**DOI:** 10.1007/s00432-014-1674-7

**Published:** 2014-05-03

**Authors:** Tomasz Wróbel, Katarzyna Gębura, Barbara Wysoczańska, Bożena Jaźwiec, Olga Dobrzyńska, Grzegorz Mazur, Kazimierz Kuliczkowski, Katarzyna Bogunia-Kubik

**Affiliations:** 1 Department of Haematology, Blood Neoplasms and Bone Marrow Transplantation, Wrocław Medical University, Wybrzeże L. Pasteura 4, 50-367 Wrocław, Poland; 2 Laboratory of Clinical Immunogenetics and Pharmacogenetics, L. Hirszfeld Institute of Immunology and Experimental Therapy, Polish Academy of Sciences, R. Weigla 12, 53-114 Wrocław, Poland; 3 Department of Internal, Occupational Diseases and Hypertension, Wrocław Medical University, Borowska 213, 50-556 Wrocław, Poland

**Keywords:** IL-17, Gene polymorphism, Disease association, Acute myeloid leukemia

## Abstract

**Purpose:**

Recent studies have suggested that Th17 cells may play a role in the pathogenesis of acute myeloid leukemia (AML). This subset of CD4+ cells is characterized by interleukin (IL)-17A and IL-17F production, which share strong homology, and surface expression of the IL-23 receptor (IL-23R). The present study aimed to determine the association between the polymorphic features located within the *IL*-*17A*, *IL*-*17F* and *IL*-*23R* genes and disease susceptibility, progression and response to therapy. In addition, the relationship between the polymorphic variants and the plasma IL-17 levels in patients was analyzed.

**Methods:**

For this purpose, 187 individuals of Polish origin including 62 AML patients and 125 healthy controls were typed for *IL*-*17A* (rs2275913; G-197A), *IL*-*17F* (rs763780; A7488G; His161Arg) and *IL*-*23R* (rs11209026, G1142A; Arg381Gln) alleles.

**Results:**

The rs763780 *IL*-*17F* polymorphism appeared to be associated with susceptibility to the disease. The presence of the minor (*G*) variant (RR = 4.76, *p* < 0.001) and its homozygosity (RR = 23.02, *p* < 0.005) was more frequent among patients than healthy individuals. No significant association was observed for either other polymorphisms studied or IL-17 levels.

**Conclusions:**

Thus, the rs763780 *IL*-*17F* polymorphism was found to be associated with predisposition to AML in the Polish population.

## Introduction

Acute myeloid leukemia (AML) is a life-threatening hematopoietic stem cell neoplasm characterized by bone marrow infiltration by leukemic cells that suppress normal hematopoiesis, frequently resulting in fatal infection, bleeding or organ infiltration, with or without leukocytosis (Lowenberg et al. 1999; Estey and Dohner 2006). The etiology of AML is heterogeneous and complex, but it is widely accepted that both environmental and genetic factors play significant roles in the development of AML.

Recent studies have suggested that Th17 cells may play an important role in patients with AML [discussed by Li et al. (2012)]. It has been reported that the Th17 cell frequencies or levels of IL-17 and its related cytokines were different between normal cells and malignant AML cells, suggesting that Th17 cells might be involved in AML pathogenesis (Wu et al. 2009; Abousamra et al. 2013). It has also been observed that the increased Th17 cell frequencies were reduced when patients achieved complete remission (CR) after chemotherapy, suggesting that measurement of Th17 cell frequencies may have clinical value in evaluation of the therapeutic effect (Wu et al. 2009).

The hallmark of the Th17 subset is the production of interleukin IL-17A and IL-17F, which share strong homology, and surface expression of the IL-23 receptor (IL-23R) (Hot et al. 2011). IL-23 is essential for the differentiation of Th17 cells and plays a key role in the development of pathogenic Th17 cells that produce the cytokine IL-17, which induces the production of several pro-inflammatory cytokines, such as TNF-a and IL-6, and chemokines (Aggarwal et al. 2003; Bettelli et al. 2008; McKenzie et al. 2006). IL-23 and IL-21 induce the orphan nuclear receptor RORγt, which in synergy with STAT3 promotes IL-17 expression (Nurieva et al. 2007).

The present study aimed to determine the association between the polymorphic features located within the *IL*-*17A*, *IL*-*17F* and *IL*-*23R* genes and disease susceptibility, progression and response to therapy. For this purpose, patients with AML and healthy individuals were typed for the *IL*-*17A* (rs2275913; G-197A), *IL*-*17F* (rs763780; A7488G; His161Arg) and *IL*-*23R* (rs11209026, G1142A; Arg381Gln) alleles. In addition, the relationship between the polymorphic variants of the IL-17 genes and plasma IL-17 levels were analyzed.

## Materials and methods

### Patients and controls

Sixty-two adult patients (24 females and 38 males, median age 52 years, range 19–80 years) with AML were investigated. Patients with acute promyelocytic leukemia were excluded.

In addition 125 Polish healthy individuals of both sexes (female/male: 63/62) served as controls.

### *IL*-*17A*, *IL*-*17F* and *IL*-*23R* genotyping

Three biallelic polymorphisms were studied: *IL*-*17A* (rs2275913; G-197A), *IL*-*17F* (rs763780; A7488G; His161Arg) and *IL*-*23R* (rs11209026, G1142A).

The *IL*-*17F* (rs763780; A7488G) polymorphism was analyzed using a polymerase chain reaction restriction fragment length polymorphism (PCR–RFLP) assay, which amplified a fragment of the promoter region of the gene using primers as previously described [15] (forward: 5′-GTT CCC ATC CAG CAA GAG AC-3′ and reverse: 5′-AGC TGG GAA TGC AAA CAA AC-3′). The PCR conditions were as follows: 94 °C for 3 min; 35 cycles at 94 °C for 30 s, 60 °C for 30 s and 72 °C for 30 s; and a final elongation step at 72 °C for 7 min. The PCR products were analyzed by electrophoresis in 2 % agarose gel stained with ethidium bromide and visualized under UV light (Uvitec). The PCR products were digested with the *Nla*III restriction endonuclease (New England BioLabs Inc.) and analyzed in 2 % agarose gel. Three patterns were observed following digestion and electrophoresis: a single 412 bp fragment (individuals homozygous for the *IL*-*17F G* allele, lacking the *Nla*III site), three fragments of 412, 288 and 124 bp in length (heterozygous individuals) or two fragments of 288 and 124 bp (individuals homozygous for the *IL*-*17F A* allele).

PCR amplifications for the *IL*-*17F* gene polymorphism studies were carried out in the 2720 Thermal Cycler (Applied Biosystems, Foster City, USA).

The *IL*-*17A* (rs2275913; G-197A) and *IL*-*23R* (rs11209026, G1142A) alleles were determined by real-time PCR amplifications, and analysis of the typing results was performed using a Roche LightCycler 480 instrument. The LightSNiP (rs2275913) assay designed by TIB MOLBIOL (GmbH, Berlin, Germany) or TaqMan SNP Genotyping Assay (rs11209026) (Life Technologies) was used for detection of *IL*-*17A* and *IL*-*23R* alleles, respectively.

### Enzyme-linked immunosorbent assay (ELISA) for plasma IL-17

Plasma samples were taken from all the patients before chemotherapy was administered. In addition, 20 out of 62 patients were analyzed again after achieving CR. IL-17 levels were measured by enzyme-linked immunoassay (ELISA) (R&D Systems, USA) following the manufacturer’s instruction. Analyses and calibrations were carried out in duplicate. Intra- and interassay variations were within the range given by the manufacturer.

Minimum detectable level of IL-17 was less than 15 pg/ml. Ten samples from healthy volunteers (4 females and 6 males, median age 40 years; range 35–60 years) were evaluated as the control. The data were analyzed using the Mann–Whitney *U* test.

### Statistical analysis

Genotype and allele frequencies were compared between the study groups by the* χ* ^2^ test with Yates correction or Fisher’s exact test when necessary using Statistica 5.5 for Windows software. The odds ratio/relative risk (RR) was calculated by Haldane’s modification of Woolf’s method and the significance of its deviation from unity was estimated by Fisher’s exact test. All *p* values were corrected (pc) for the number of comparisons. The IL-17 plasma levels were analyzed using the Mann–Whitney *U* test. Probability values <0.05 were considered statistically significant and those between 0.05 and 0.1 as indicative of a trend.

## Results

### Distribution of *IL*-*17A* and *IL*-*23R* alleles and genotypes in AML patients and controls

There were no significant differences in the distributions of the *IL*-*17A* and *IL*-*23R* alleles and genotypes. The *IL*-*17A* (rs2275913) *GG*, *GA* and *AA* genotypes were detected in 23 (37.1 %), 25 (40.3 %) and 14 (22.6 %) patients, and in 38 (30.4 %), 67 (53.6 %) and 20 (16 %) controls, respectively. Only a slight tendency toward a higher frequency of the *IL*-*17A* heterozygosity was observed (RR = 1.73, *p* = 0.091, Table 1). The allelic frequencies of the *A* variant of the *IL*-*17A* gene were 0.427 and 0.428, in patients and controls, respectively, which closely resemble those observed in other studies of healthy European populations.

**Table 1:** Distribution of the *IL*-*17A*, *IL*-*17F* and *IL*-*23R* alleles and genotypes in Polish patients with AML and healthy individuals

Polymorphism	AML patients		Controls	
	*n*	(%)	*n*	(%)
*IL*-*17A* (G-197A) rs2275913				
*GG*	23	37.1	38	30.4
*GA*	25	40.3	67	53.6
*AA*	14	22.6	20	16.0
*G*	48	77.4	105	84.0
*A*	14	62.9	87	69.6
*IL*-*17F* (A7488G) rs763780				
*AA*	42	67.7	114	91.2
*AG*	15	24.2	11	8.8
*GG*	5^b^	8.1	0^b^	0
*A*	57	91.9	125	100
*G*	20^a^	32.3	11^a^	8.8
*IL*-*23R* (G1142A) rs11209026				
*GG*	55	88.70	111	88.8
*GA*	7	11.3	14	11.2
*AA*	0	0	0	0
*G*	62	100	125	100
*A*	7	11.3	14	11.2

The *IL*-*17F* polymorphism was associated with predisposition to AML

^a^RR = 4.76, *p* = 0.0009, pc = 0.0027

^b^RR = 23.02, *p* = 0.0036, pc = 0.0108

The *IL*-*23R A* variant was very rarely detected. None of the individuals tested was carrying the *IL*-*23R AA* homozygous genotype. Fifty-five patients (88.7 %) and 111 (88.8 %) healthy individuals were homozygous for the *IL*-*23R* (rs11209026) *G* wild-type allele. There were only 7 (11.3 %) and 14 (11.2 %) *GA* heterozygotes among AML patients and controls, respectively (Table 1). The allelic frequency of the *A* variant was 0.056 in both patients and controls.

### Associations with predisposition to AML and progression of the disease, impact of the *IL*-*17F* polymorphism

Among the polymorphisms studied, the *IL*-*17F* (A7488G) polymorphic variants were found to be associated with AML. The presence of the *IL*-*17F G* allele was more frequently observed among patients than healthy individuals. This allelic variant was detected in 20 out of 62 (32.3 %) patients with AML and only in 11 out of 125 (8.8 %) controls (RR = 4.76, pc < 0.001). The allelic frequencies of the *G* variant of the *IL*-*17F* gene were 0.202 and 0.044, in patients and controls, respectively.

**Fig. 1:**
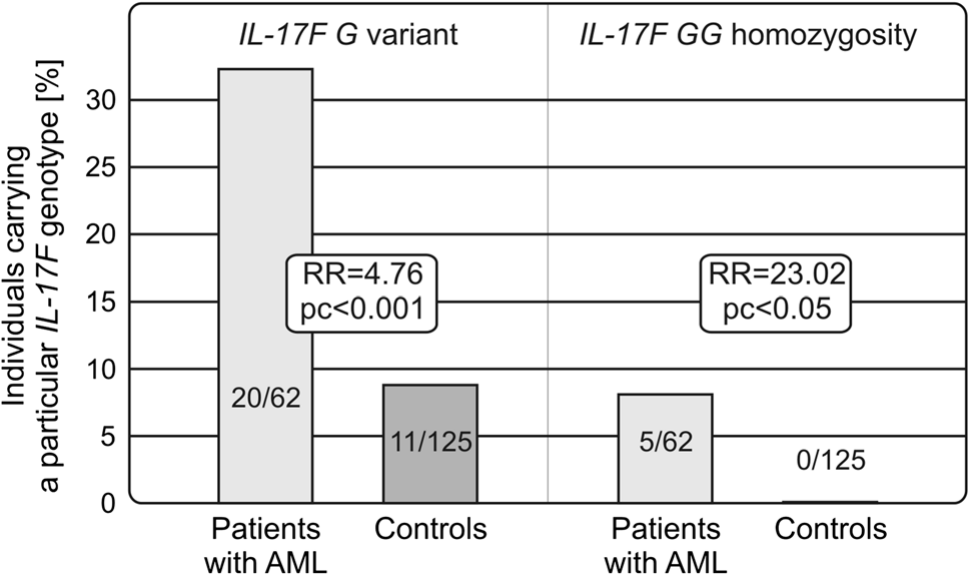
Associations of the *IL*-*17F* (rs763780; A7488G) polymorphism with susceptibility to AML. The *IL*-*17F 7488G* variant and *GG* homozygosity were more frequently detected in patients with AML

An even stronger association was observed when the *IL*-*17F GG* homozygous genotype was considered. *IL*-*17F GG* homozygosity was detected in five patients and none of the controls (RR = 23.02, pc < 0.05; Table 1; Fig. 1).

### Relationships between the *IL*-*17F* polymorphic variants and IL-17 plasma levels

IL-17 was detected in 9 out of 62 AML patients at diagnosis (range, mean ± SD: 1.37–19.10, 8.8 ± 7.19 pg/ml), 2 out of 19 in AML in CR, and 2 out of 10 in the control group (at the level of 1.4 pg/ml in these 4 latter samples).

No significant relationships were observed with respect to the IL-17 plasma levels and disease progression (CR) or any of the polymorphisms studied.

### No correlation between the polymorphisms studied and response to therapy or survival

None of the polymorphisms studies were observed to be associated with response to therapy or patient survival. All genotypes segregated similarly in alive and fatal cases. There was no significant difference in the distribution of *IL*-*17A*, *IL*-*17F* and *IL*-*23R* genotypes between patients who achieved or did not achieve CR.

## Discussion

In the present study, we wanted to find out whether polymorphic variants of the genes coding for IL-17A, IL-17F and receptor for IL-23 (as hallmarks of Th17 cells) could be associated with susceptibility to acute myeloid leukemia, disease progression and/or response to therapy. In addition, the relationship between the polymorphic variants of the IL-17 encoding genes and the plasma IL-17 levels was analyzed.

Among the three biallelic polymorphisms considered, only the *IL*-*17F* (A7488G) polymorphism was found to be associated with predisposition to AML. The presence of the *IL*-*17F G* variant and its homozygosity were significantly more frequently observed among patients than healthy individuals. This is a novel observation not previously described.

Interestingly, Kawaguchi et al. observed the functional consequences of this *IL*-*17F* polymorphism and suggested that the IL-17 expression and activity may be suppressed in carriers of the rare *G* allele (Kawaguchi et al. 2006). However, this effect was not observed in our present study. As suggested by Kawaguchi et al., we might expect that patients carrying this allelic variant would be characterized by lower levels of IL-17. However, we found no significant differences in the IL-17 plasma levels between AML patients with different *IL*-*17F* genotypes.

As for the rs2275913 *IL*-*17A* polymorphism, Espinoza et al. demonstrated that in vitro stimulated T cells from healthy individuals possessing the *197A* allele produced significantly more IL-17 than those without the *197A* allele (Espinoza et al. 2011). However, similarly as for the *IL*-*17F* polymorphism, no significant relationships between the *IL*-*17A* genotypes and IL-17 plasma levels were observed in the present study.

Moreover, as previously found, no difference was detected between patients and controls with respect to the IL-17 plasma levels (Wrobel et al. 2003).

Notably, the differences in IL-17 concentrations and frequencies of IL-17 producing cells have been previously reported in the literature, suggesting that their increase may have an adverse effect. For example, Wu et al. observed significantly increased frequencies of Th17 cells in peripheral blood samples from untreated patients with AML, compared with those from healthy volunteers (Wu et al. 2009). They also found that increased IL-17 concentrations accompanied the increased Th17 cell frequencies in these patients and that the increased Th17 cell frequencies were reduced when patients achieved CR after chemotherapy. In a more recent report of Abousamra et al., who studied patients with acute myeloid (AML) and acute lymphoblastic leukemia (ALL), circulating Th17 cells were increased in patients with acute leukemias and were significantly higher than in healthy controls. In these patients, Th17 cells were reduced significantly in those who achieved CR after induction therapy (Abousamra et al. 2013). Also Tian et al. observed higher frequencies of Th17 cells and a significant decrease in IL-17 concentration in patients with T-ALL (both newly diagnosed and in CR) than in controls (Tian et al. 2013).

It is of note that the results of the three latter studies consider patients of Chinese (Wu et al. 2009; Tian et al. 2013) or Egyptian (Abousamra et al. 2013) origin. Thus, the differences in the results could be attributed to the compositions of patient groups studied, including the lack of an association between the *IL*-*17F* variants and Il-17 plasma levels [reported by Kawaguchi et al. (2006) for the Japanese population].

To our knowledge, there are no published data on the role of the *IL*-*17A*, *IL*-*17F* or *IL*-*23R* polymorphisms in AML; thus, our report presents novel observations not previously described. We found that they significantly contribute to the disease susceptibility in Polish patients with AML. Obviously, these results should be confirmed in a more extended study, including patients from other centers.
